# Whole Aspect of Runx2 Functions in Skeletal Development

**DOI:** 10.3390/ijms23105776

**Published:** 2022-05-21

**Authors:** Toshihisa Komori

**Affiliations:** Department of Molecular Bone Biology, Nagasaki University Graduate School of Biomedical Sciences, Nagasaki 852-8588, Japan; komorit@nagasaki-u.ac.jp; Tel.: +81-95-819-7637; Fax: +81-95-819-7638

**Keywords:** Runx2, chondrocyte differentiation, osteoblast proliferation, osteoblast differentiation, transdifferentiation, bone matrix proteins, Ihh, Fgfr, Wnt, Sp7

## Abstract

Runt-related transcription factor 2 (Runx2) is a fundamental transcription factor for bone development. In endochondral ossification, Runx2 induces chondrocyte maturation, enhances chondrocyte proliferation through Indian hedgehog (Ihh) induction, and induces the expression of vascular endothelial growth factor A (Vegfa), secreted phosphoprotein 1 (Spp1), integrin-binding sialoprotein (Ibsp), and matrix metallopeptidase 13 (Mmp13) in the terminal hypertrophic chondrocytes. Runx2 inhibits the apoptosis of the terminal hypertrophic chondrocytes and induces their transdifferentiation into osteoblasts and osteoblast progenitors. The transdifferentiation is required for trabecular bone formation during embryonic and newborn stages but is dispensable for acquiring normal bone mass in young and adult mice. Runx2 enhances the proliferation of osteoblast progenitors and induces their commitment to osteoblast lineage cells through the direct regulation of the expressions of a hedgehog, fibroblast growth factor (Fgf), Wnt, and parathyroid hormone-like hormone (Pthlh) signaling pathway genes and distal-less homeobox 5 (Dlx5), which all regulate Runx2 expression and/or protein activity. Runx2, Sp7, and Wnt signaling further induce osteoblast differentiation. In immature osteoblasts, Runx2 regulates the expression of bone matrix protein genes, including Col1a1, Col1a2, Spp1, Ibsp, and bone gamma carboxyglutamate protein (Bglap)/Bglap2, and induces osteoblast maturation. Osteocalcin (Bglap/Bglap2) is required for the alignment of apatite crystals parallel to the collagen fibers; however, it does not physiologically work as a hormone that regulates glucose metabolism, testosterone synthesis, or muscle mass. Thus, Runx2 exerts multiple functions essential for skeletal development.

## 1. Introduction

Runt-related transcription factor 2 (Runx2) is a transcription factor that belongs to the Runx family, which is composed of Runx1, Runx2, and Runx3 [1]. Runx2 is expressed in chondrocytes, osteoblast lineage cells, and thymocytes. Runx2 is essential for chondrocyte maturation and osteoblast differentiation, but not for T cell development [2]. Although Runx1 and Runx3 are majorly involved in oncogenesis, Runx1 is partly involved in sternal morphogenesis, and Runx3 is involved in chondrocyte maturation and osteoblast proliferation [3,4,5,6]. Cotranscription factor core binding factor β (Cbfb) heterodimerizes with the Runx family proteins, stabilizes them, and enhances their DNA binding capacity [7,8,9,10,11,12,13,14,15,16]. Generally, the unclarified aspects of Runx2 functions are being resolved. This review will focus on the Runx2 functions in skeletal development.

## 2. Runx2 and Chondrocyte Differentiation

**Figure 1:**
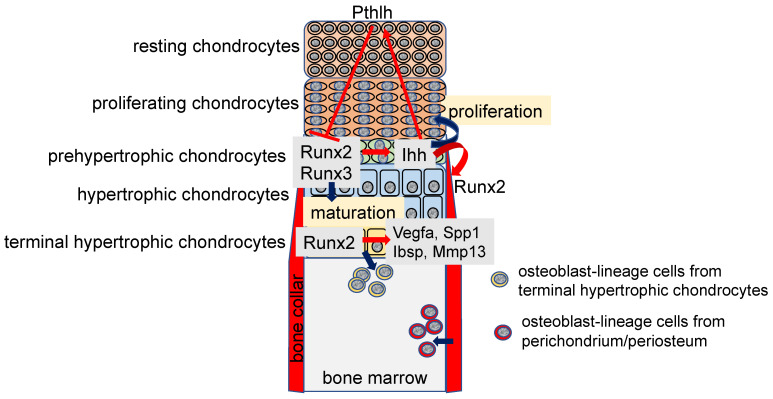
Regulation of chondrocyte maturation and proliferation in the growth plate and osteoblastogenesis in the bone marrow: Runt-related transcription factor 2 (Runx2) expression is upregulated in prehypertrophic chondrocytes and Runx2 induces chondrocyte maturation. Runx3 is also involved in chondrocyte maturation. Runx2 induces Indian hedgehog (Ihh) expression in the prehypertrophic chondrocytes, and Ihh enhances the proliferation of chondrocytes in the proliferating layer. Ihh also induces the expression of parathyroid hormone like hormone (Pthlh), which inhibits Runx2 expression through parathyroid hormone 1 receptor (Pth1r), and inhibits chondrocyte maturation, forming a negative feedback loop of chondrocyte maturation. Ihh induces Runx2 expression and osteoblast differentiation in the perichondrium. Runx2 induces the expressions of vascular endothelial growth factor A (Vegfa), secreted phosphoprotein 1 (Spp1), integrin binding sialoprotein (Ibsp), and matrix metallopeptidase 13 (Mmp13) in the terminal hypertrophic chondrocytes. Runx2 inhibits the apoptosis of terminal hypertrophic chondrocytes and induces the transdifferentiation of the terminal hypertrophic chondrocytes into osteoblast lineage cells. Osteoblasts in the bone marrow are derived from chondrocytes in the growth plate and osteoblast progenitors in the perichondrium/periosteum.

In the growth plate, chondrocytes proliferate and maturate, making the resting, proliferating, prehypertrophic, hypertrophic, and terminal hypertrophic layers (Figure 1). Chondrocytes in the resting and proliferating layers are immature, actively proliferate, and highly express Col2a1. Those in the prehypertrophic layer express parathyroid hormone 1 receptor (Pth1r) and Indian hedgehog (Ihh), those in the hypertrophic layer express Col10a1, and those in the terminal hypertrophic layer express vascular endothelial growth factor A (Vegfa), secreted phosphoprotein 1 (Spp1), integrin-binding sialoprotein (Ibsp), and matrix metallopeptidase 13 (Mmp13) [17]. Runx2 is weakly expressed in chondrocytes in the resting and proliferation layers and upregulated in the prehypertrophic chondrocytes, and the expression is maintained until terminal differentiation of hypertrophic chondrocytes [17,18] (Figure 1). Chondrocyte maturation is markedly impaired in Runx2-deficient (Runx2^−/−^) mice, which exhibit an absence of hypertrophic chondrocytes in most of the skeleton, except for the tibia, fibula, radius, and ulna. Overexpression of Runx2 under the control of the Col2a1 promoter, which directs the transgene to chondrocytes, accelerates chondrocyte maturation and endochondral ossification, and chondrocytes in the presumptive permanent cartilage, including articular cartilage, also enter the process of endochondral ossification [19,20]. In contrast, as overexpression of dominant-negative Runx2 under the control of the Col2a1 promoter decelerates chondrocyte maturation, most of the endochondral skeletons are composed of immature chondrocytes, and the immature chondrocytes express tenascin-C (Tnc), which is expressed in the permanent cartilage of wild-type mice [19]. Conditional knockout mice using Col2a1 Cre transgenic mice to delete Runx2 in cartilage (Col2a1 Cre Runx2^fl/fl^ mice) also exhibit inhibition of chondrocyte maturation [21,22].

Runx3 is expressed in prehypertrophic and hypertrophic chondrocytes, and chondrocyte maturation is slightly delayed in Runx3^−/−^ embryos. Furthermore, Runx2^−/−^Runx3^−/−^ mice show a complete absence of chondrocyte maturation, indicating that Runx3 is also involved in chondrocyte maturation, and Runx2 and Runx3 are essential for chondrocyte maturation [5] (Figure 1). Runx2 is involved in chondrocyte proliferation by directly regulating the expression of Ihh, which enhances chondrocyte proliferation [5,23] (Figure 1). In addition, Runx2 interacts with doublesex and mab-3 related transcription factor 2 (Dmrt2) and regulates chondrocyte maturation and Ihh expression [24]. As Ihh induces parathyroid hormone-like hormone (Pthlh), which is expressed in the epiphysis and inhibits chondrocyte maturation by suppressing Runx2 expression through Pth1r, a negative feedback loop of Runx2-Ihh-Pthlh-Pth1r is formed in the regulation of chondrocyte maturation by Runx2 [25,26,27] (Figure 1).

## 3. Functions of Runx2 in Terminal Hypertrophic Chondrocytes

As chondrocyte hypertrophy is inhibited in Runx2^−/−^ mice, dominant-negative Runx2 transgenic mice under the control of Col2a1 promoter, and Col2a1 Cre Runx2^fl/fl^ mice [17,18,19,21,22], the function of Runx2 in the terminal hypertrophic chondrocytes was not clear. After chondrocyte maturation, the vascular invasion occurs at the layer of the terminal hypertrophic chondrocytes, which express Vegfa. As vascular invasion into the cartilage did not occur in Runx2^−/−^ mice and Vegfa expression was reduced in the terminal hypertrophic chondrocytes, the induction of Vegfa expression by Runx2 in the terminal hypertrophic chondrocytes was considered to be required for the vascular invasion into the cartilage [17,18,28,29]. In Runx2 conditional knockout mice using Col10a1 Cre transgenic mice (Col10a1 Cre Runx2^fl/fl^ mice) to delete Runx2 in hypertrophic chondrocytes, chondrocyte maturation to terminal hypertrophic chondrocytes and vascular invasion into the cartilage occurred normally, although Vegfa expression in the terminal hypertrophic chondrocytes was reduced [30]. Moreover, Vegfa is strongly expressed in osteoblasts [30]. Ihh, which is expressed in the prehypertrophic chondrocytes, induces Runx2 expression and osteoblast differentiation in the perichondrium, leading to the formation of the bone collar [23,31] (Figure 1). As Ihh was normally expressed in the prehypertrophic chondrocytes of Col10a1 Cre Runx2^fl/fl^ mice, osteoblast differentiation in the perichondrium and bone collar formation normally occurred, and Vegfa was strongly expressed in the osteoblasts. Thus, Vegfa expression in osteoblasts in the bone collar is sufficient for vascular invasion into the cartilage [30].

Spp1 and Ibsp, which are noncollagenous matrix proteins, are expressed in terminal hypertrophic chondrocytes and osteoblasts, and Mmp13, which is a matrix metalloproteinase that cleaves type II collagen over type I and III, is expressed in terminal hypertrophic chondrocytes [30,32]. Spp1 and Mmp13 have been shown to be regulated by Runx2 in vitro [33,34,35,36,37,38]. Indeed, the expressions of Spp1, Ibsp, and Mmp13 are markedly reduced in Runx2^−/−^ mice due to the inhibited hypertrophy and absence of osteoblasts [17]. Therefore, the regulation of Spp1, Ibsp, and Mmp13 expression in terminal hypertrophic chondrocytes by Runx2 remains to be clarified. Although terminal differentiation of chondrocytes in Col10a1 Cre Runx2^fl/fl^ mice occurred normally, the expressions of Spp1, Ibsp, and Mmp13 were reduced in the terminal hypertrophic chondrocytes [30]. Thus, the expressions of Vegfa, Spp1, Ibsp, and Mmp13 in the terminal hypertrophic chondrocytes are regulated by Runx2 in vivo (Figure 1).

## 4. Runx2 and the Transdifferentiation of Chondrocytes into Osteoblasts

Although terminal hypertrophic chondrocytes were considered to die by apoptosis, cell-lineage tracing experiments using Col10a1 Cre transgenic mice revealed that the terminal hypertrophic chondrocytes in the growth plate transdifferentiate into osteoblasts or osteoblast progenitors [39,40,41,42]. Furthermore, Pthlh-positive chondrocytes in the resting layer of the growth plate and borderline chondrocytes, which are located adjacent to the perichondrium, become stromal cells and osteoblasts through the Col10a1-positive stage [43,44]. In Col10a1 Cre Runx2^fl/fl^ mice, the transdifferentiation was interrupted, and the frequency of apoptosis of the terminal hypertrophic chondrocytes increased. Therefore, Runx2 is required for the transdifferentiation and survival of the terminal hypertrophic chondrocytes [30] (Figure 1). The trabecular bone formation was delayed in Col10a1 Cre Runx2^fl/fl^ embryos and newborn mice. Although the frequencies of the transdifferentiated osteoblasts in the trabecular bone and endosteum of cortical bone in femurs or tibiae ranged from 15 to 60% depending on the reports [30,40,41], Col10a1 Cre Runx2^fl/fl^ mice between 6 and 20 weeks old had similar bone volume and bone formation rate in the trabecular and cortical bone of femurs compared with those in wild-type mice of the same age [30]. Thus, transdifferentiation is required for trabecular bone formation during embryonic and newborn stages but is dispensable for acquiring normal bone mass in young and adult mice; osteoblasts derived from perichondrium/periosteum are likely to be a major source of osteoblasts after birth (Figure 1).

## 5. The Commitment of Mesenchymal Cells to an Osteoblastic Lineage

**Figure 2:**
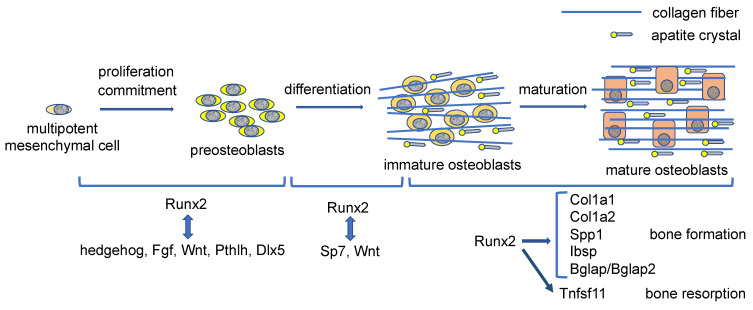
Regulation of proliferation, differentiation, and bone matrix protein gene expression by Runx2 during osteoblast differentiation: Runx2 induces the commitment of multipotent mesenchymal cells to preosteoblasts and enhances the proliferation of osteoblast progenitors by inducing the expressions of hedgehog, fibroblast grow factor (Fgf), Wnt, and Pthlh signaling pathway genes and distal-less homeobox 5 (Dlx5), which also enhance Runx2 expression and Runx2 protein activity. Runx2 also induces Sp7 expression. Runx2, Sp7 and Wnt signaling induce the differentiation of preosteoblasts into immature osteoblasts. Sp7 and Wnt signaling activate the osteoblast-specific Runx2 enhancer. In immature osteoblasts, Runx2 induces the expressions of bone matrix protein genes, including Col1a1, Col1a2, Spp1, Ibsp, and bone gamma carboxyglutamate protein (Bglap)/Bglap2, and the osteoblasts maturate and produce abundant bone matrix proteins. Runx2 also induces tumor necrosis factor superfamily member 11 (Tnfsf11) expression and enhances bone resorption. Osteocalcin (Bglap/Bglap2) aligns the apatite crystals parallel to collagen fibers. The collagen fibers run along the longitudinal direction in the dialysis of long bone, which is formed by mature osteoblasts, whereas collagen fibers run irregularly in metaphyseal cortical bone in young mice, which is formed by immature osteoblasts. However, apatite crystals are always aligned parallel to the collagen fibers by osteocalcin [63].

Ihh expression in the prehypertrophic chondrocytes is required for Runx2 expression and osteoblast differentiation in the perichondrium [23] (Figure 1). Sp7^−/−^ mice show a lack of osteoblasts, demonstrating that Sp7 is an essential transcription factor for osteoblast differentiation [45]. Catenin beta 1 (Ctnnb1) in the nucleus heterodimerizes with T cell factor (TCF)/lymphoid enhancer-binding factor (LEF) transcription factors and induces Wnt target gene expression. As Ctnnb1^−/−^ mice exhibit a lack of osteoblasts, Wnt signaling is also essential for osteoblast differentiation [46,47,48,49]. Although Runx2^−/−^ mice also completely lack osteoblasts, Runx2 is expressed in the mesenchymal cells in Sp7^−/−^ mice and Ctnnb1^−/−^ mice, indicating that Runx2 works upstream of Sp7 and Wnt signaling in osteoblast differentiation [45,46,47,48,49,50,51]. Distal-less homeobox 5 (Dlx5) is a homeobox gene expressed in osteoblasts and plays an important role in bone development because Dlx5^−/−^ mice show delayed mineralization [52]. Haplodeficiency of RUNX2 in humans causes cleidocranial dysplasia, which is characterized by open fontanelles and sutures, hypoplastic clavicles, supernumerary teeth, and short stature [53,54]. Although open fontanelles and sutures and hypoplastic clavicles are prominent skeletal abnormalities in Runx2^+/−^ mice, the reasons for the severe phenotypes in the calvaria and clavicles remain to be clarified. The frequency of proliferation of mesenchymal cells in the sutures in Runx2^+/−^ mice is low, and the expression of hedgehog {Gli1, patched 1 (Ptch1), Ihh}, Fgf {Fgf receptor 2 (Fgfr2), Fgfr3}, Wnt {transcription factor 7 (Tcf7), Wnt10b}, and Pthlh (Pth1r) signaling pathway genes and Dlx5, all of which are directly regulated by Runx2, is reduced in the sutures [55]. However, these gene expressions are not reduced in calvarial bone tissues in Runx2^+/−^ mice. Consequently, more than a half dosage of Runx2 is required for the induction of these signaling pathway genes and Dlx5 in mesenchymal cells in sutures but not in osteoblasts. These signaling pathways induce the proliferation of mesenchymal cells in sutures and induce osteoblast differentiation and bone formation. Therefore, Runx2 induces the proliferation of mesenchymal cells and induces their commitment to osteoblast lineage cells by regulating these signaling pathway genes and Dlx5 [55] (Figure 2). The Fgf signaling pathway activates and stabilizes Runx2 protein through phosphorylation by mitogen-activated protein kinase 1/3 (Mapk1/3); Tcf7, Ctnnb1, and Dlx5 activate the promoter and osteoblast-specific enhancer of Runx2; parathyroid hormone (Pth) increases Runx2 activity and Runx2 mRNA [36,56,57,58,59,60,61]. Thus, these signaling pathway genes and Dlx5 enhance Runx2 expression and activity, making a reciprocally regulatory network (Figure 2). Runx2 also induces Sp7 expression, and Runx2, Sp7, and Wnt signaling induce the differentiation of preosteoblasts into immature osteoblasts [45,46,47,48,49,62] (Figure 2). Sp7 is also involved in the activation of osteoblast-specific Runx2 enhancers [59].

## 6. Runx2 and Proliferation of Osteoblast Progenitors

Mesenchymal cells derived from the presumptive calvaria of Runx2^−/−^ embryos proliferate faster than osteoblastic cells derived from wild-type calvaria in vitro [64]. Therefore, Runx2 was considered to inhibit the proliferation of osteoblast progenitors. However, the number of mesenchymal cells in the presumptive calvaria, mandible, and limbs in Runx2^−/−^ embryos is extremely low. Although there are no osteoblasts in the whole skeleton of Sp7^−/−^ embryos and Runx2^−/−^ embryos, Sp7^−/−^ embryos have sufficient preosteoblasts in the calvaria, mandible, and limbs, in which Runx2 is expressed [65]. Furthermore, the amount of the preosteoblasts in Sp7^−/−^Runx2^+/−^ embryos is half that of Sp7^−/−^ embryos, indicating that the amount of the preosteoblasts in Sp7^−/−^ embryos are dependent on the gene dosage of Runx2. Runx2 induces the proliferation of osteoblast progenitors and enhances the proliferation induced by Fgf2. In addition, Runx2 directly regulates the expression of Fgfr2 and Fgfr3 [65]. Thus, Runx2 is required for the proliferation of osteoblast progenitors and induces it, at least in part, through the induction of Fgfr2 and Fgfr3 expression (Figure 2).

## 7. Runx2 Functions in Differentiated Osteoblasts

Runx2 induces the expression of bone matrix protein genes, including Col1a1, Spp1, Ibsp, and Bglap/Bglap2, in vitro [66]. However, the phenotypes of Runx2 conditional knockout mice using Cre transgenic mice under the control of the 2.3 kb Col1a1 promoter, which directs the Cre expression in differentiated osteoblasts, were controversial in two groups, although they used the same Col1a1 Cre transgenic mouse line [21,67]. The deletion of the runt domain, which is essential for DNA binding, in osteoblasts resulted in no phenotypes [21], whereas the deletion of exon 8, which creates a cryptic Runx2 protein that maintains DNA binding capacity but has a lower capacity for transcriptional activation, in osteoblasts results in reduced bone mass [67]. The cryptic Runx2 protein may have interrupted the binding of Runx3, which regulates osteoblast proliferation [6].

Runx2 conditional knockout mice have been generated by deleting the runt domain using newly generated 2.3 kb Col1a1 Cre transgenic mice [68]. The body weight was lower, clavicles were shorter, the unmineralized area in calvaria was wider, and incisors were shorter in the Runx2 conditional knockout mice compared with those in the control mice at 6 weeks of age. The trabecular bone volume was lower and cortical bone was thinner in femurs; the bone formation was lower in the trabecular and cortical bone; the bone volume of vertebrae was lower, and the serum markers for bone formation and resorption were lower in the Runx2 conditional knockout mice than those in the control mice. These observations were seen in both males and females. Furthermore, osteoblast proliferation was reduced in the Runx2 conditional knockout mice. The expressions of bone matrix protein genes, including Col1a1, Col1a2, Spp1, Ibsp, and Bglap/Bglap2, were reduced in the Runx2 conditional knockout mice and the osteoblasts had a lower amount of cytoplasm compared with those in control mice, indicating that Runx2 is required for the major bone matrix protein gene expression (Figure 2). Moreover, the expressions of Runx2 target genes, including Ihh, Ptch1, Fgfr1, Fgfr2, Fgfr3, Tcf7, Wnt10b, Pth1r, Sp7, and Dlx5, were also reduced. Thus, Runx2 regulates Ihh, Fgf, Wnt, and Pthlh signaling pathways and the expression of transcription factors, Sp7 and Dlx5, after differentiation into osteoblasts and during osteoblast differentiation. As hedgehog and Wnt signaling and Sp7 are also involved in bone formation by mature osteoblasts [69,70,71,72], Runx2 regulates bone formation not only through the direct regulation of bone matrix protein gene expression but also through the regulation of these target gene expressions. The proteins transcribed by these target genes also regulate Runx2 gene transcription and Runx2 protein activity and stability [36,56,57,58,59,60,61]. In addition, Runx2 enhances bone resorption through the regulation of Tnfsf11 [68,73] (Figure 2).

Osteocalcin is the most abundant non-collagenous bone matrix protein and is transcribed from Bglap and Bglap2, whose expressions are directly regulated by Runx2 [63]. Bglap/Bglap2^−/−^ mice are normal in bone mass, bone formation, and bone resorption [74,75]. Although collagen fibers run along the longitudinal direction of the long bone in wild-type and Bglap/Bglap2^−/−^ mice, the orientation of apatite crystals, which is parallel to collagen fibers in wild-type mice, is markedly disrupted in Bglap/Bglap2^−/−^ mice, and the bone strength is reduced [74]. Therefore, osteocalcin is required for the alignment of apatite crystals parallel to collagen fibers (Figure 2). Although osteocalcin has been shown to regulate glucose metabolism, testosterone synthesis, and muscle mass, these are normal in Bglap/Bglap2^−/−^ mice [74,75]. Thus, osteocalcin is important for bone quality but does not work as a hormone [63].

## 8. Conclusions

Runx2 has multiple functions in skeletal development, including chondrocyte maturation, osteoblast differentiation, the proliferation of chondrocytes and osteoblast progenitors, transdifferentiation of chondrocytes, and matrix protein gene expression in chondrocytes and osteoblasts. Runx2 is a good target for the treatment of osteoporosis by increasing bone formation. As Runx2 has multiple functions in osteoblasts and chondrocytes, however, it is important to specifically upregulate Runx2 expression or activity in osteoblast lineage cells. The upregulation of Runx2 in the articular cartilage will lead to osteoarthritis due to the maturation of chondrocytes and degradation of the cartilage matrix [19,76]. In contrast, the suppression of Runx2 in the articular cartilage will be beneficial for osteoarthritis [77,78,79]. Thus, the lineage-specific regulation of Runx2 is required for clinical application. 

## Data Availability

No available data.

## References

[1] Komori T. (2019). Regulation of Proliferation, Differentiation and Functions of Osteoblasts by Runx2. Int. J. Mol. Sci..

[2] Komori T. (2005). Regulation of skeletal development by the Runx family of transcription factors. J. Cell. Biochem..

[3] Ito Y., Bae S.-C., Chuang L.S.H. (2015). The RUNX family: Developmental regulators in cancer. Nat. Cancer.

[4] Kimura A., Inose H., Yano F., Fujita K., Ikeda T., Sato S., Iwasaki M., Jinno T., Ae K., Fukumoto S. (2010). Runx1 and Runx2 cooperate during sternal morphogenesis. Development.

[5] Yoshida C.A., Yamamoto H., Fujita T., Furuichi T., Ito K., Inoue K., Yamana K., Zanma A., Takada K., Ito Y. (2004). Runx2 and Runx3 are essential for chondrocyte maturation, and Runx2 regulates limb growth through induction of Indian hedgehog. Genes Dev..

[6] Bauer O., Sharir A., Kimura A., Hantisteanu S., Takeda S., Groner Y. (2015). Loss of Osteoblast Runx3 Produces Severe Congenital Osteopenia. Mol. Cell. Biol..

[7] Kundu M., Javed A., Jeon J.P., Horner A., Shum L., Eckhaus M., Muenke M., Lian J.B., Yang Y., Nuckolls G.H. (2002). Cbfβ interacts with Runx2 and has a critical role in bone development. Nat. Genet..

[8] Miller J., Horner A., Stacy T., Lowrey C., Lian J.B., Stein G., Nuckolls G.H., Speck N.A. (2002). The core-binding factor β subunit is required for bone formation and hematopoietic maturation. Nat. Genet..

[9] Yoshida C.A., Furuichi T., Fujita T., Fukuyama R., Kanatani N., Kobayashi S., Satake M., Takada K., Komori T. (2002). Core-binding factor β interacts with Runx2 and is required for skeletal development. Nat. Genet..

[10] Chen W., Ma J., Zhu G., Jules J., Wu M., McConnell M., Tian F., Paulson C., Zhou X., Wang L. (2014). Cbfβ deletion in mice recapitulates cleidocranial dysplasia and reveals multiple functions of Cbfβ required for skeletal development. Proc. Natl. Acad. Sci. USA.

[11] Tian F., Wu M., Deng L., Zhu G., Ma J., Gao B., Wang L., Li Y.-P., Chen W. (2014). Core binding factor β (Cbfβ) controls the balance of chondrocyte proliferation and differentiation by upregulating Indian hedgehog (Ihh) expression and inhibiting parathyroid hormone-related protein receptor (PPR) expression in postnatal cartilage and bone formation. J. Bone Miner. Res..

[12] Lim K.E., Park N.R., Che X., Han M.S., Jeong J.H., Kim S.Y., Park C.Y., Akiyama H., Kim J.E., Ryoo H.M. (2015). Core binding factor β of osteoblasts maintains cortical bone mass via stabilization of Runx2 in mice. J. Bone Miner. Res..

[13] Qin X., Jiang Q., Matsuo Y., Kawane T., Komori H., Moriishi T., Taniuchi I., Ito K., Kawai Y., Rokutanda S. (2014). Cbfb Regulates Bone Development by Stabilizing Runx Family Proteins. J. Bone Miner. Res..

[14] Jiang Q., Qin X., Kawane T., Komori H., Matsuo Y., Taniuchi I., Ito K., Izumi S.-I., Komori T. (2016). Cbfb2 Isoform Dominates More Potent Cbfb1 and Is Required for Skeletal Development. J. Bone Miner. Res..

[15] Ogawa E., Inuzuka M., Maruyama M., Satake M., Naito-Fujimoto M., Ito Y., Shigesada K. (1993). Molecular cloning and characterization of PEBP2 β, the heterodimeric partner of a novel Drosophila runt-related DNA binding protein PEBP2 α. Virology.

[16] Wang S., Wang Q., Crute B.E., Melnikova I.N., Keller S.R., Speck N.A. (1993). Cloning and characterization of subunits of the T-cell receptor and murine leukemia virus enhancer core-binding factor. Mol. Cell. Biol..

[17] Inada M., Yasui T., Nomura S., Miyake S., Deguchi K., Himeno M., Sato M., Yamagiwa H., Kimura T., Yasui N. (1999). Maturational disturbance of chondrocytes in Cbfa1-deficient mice. Dev. Dyn..

[18] Kim I., Otto F., Zabel B., Mundlos S. (1999). Regulation of chondrocyte differentiation by Cbfa1. Mech. Dev..

[19] Ueta C., Iwamoto M., Kanatani N., Yoshida C., Liu Y., Enomoto-Iwamoto M., Ohmori T., Enomoto H., Nakata K., Takada K. (2001). Skeletal Malformations Caused by Overexpression of Cbfa1 or Its Dominant Negative Form in Chondrocytes. J. Cell Biol..

[20] Takeda S., Bonnamy J.-P., Owen M.J., Ducy P., Karsenty G. (2001). Continuous Expression of Cbfa1 in Nonhypertrophic Chondrocytes Uncovers Its Ability to Induce Hypertrophic Chondrocyte Differentiation and Partially Rescues Cbfa1-Deficient Mice. Genes Dev.

[21] Takarada T., Hinoi E., Nakazato R., Ochi H., Xu C., Tsuchikane A., Takeda S., Karsenty G., Abe T., Kiyonari H. (2013). An analysis of skeletal development in osteoblast-specific and chondrocyte-specific runt-related transcription factor-2 (Runx2) knockout mice. J. Bone Miner. Res..

[22] Chen H., Ghori-Javed F.Y., Rashid H., Adhami M.D., Serra R., Gutierrez S.E., Javed A. (2014). Runx2 Regulates Endochondral Ossification Through Control of Chondrocyte Proliferation and Differentiation. J. Bone Miner. Res..

[23] St-Jacques B., Hammerschmidt M., McMahon A.P. (1999). Indian hedgehog signaling regulates proliferation and differentiation of chondrocytes and is essential for bone formation. Genes Dev..

[24] Ono K., Hata K., Nakamura E., Ishihara S., Kobayashi S., Nakanishi M., Yoshida M., Takahata Y., Murakami T., Takenoshita S. (2021). Dmrt2 promotes transition of endochondral bone formation by linking Sox9 and Runx2. Commun. Biol..

[25] Iwamoto M., Kitagaki J., Tamamura Y., Gentili C., Koyama E., Enomoto H., Komori T., Pacifici M. (2003). Runx2 expression and action in chondrocytes are regulated by retinoid signaling and parathyroid hormone-related peptide (PTHrP). Osteoarthr. Cartil..

[26] Li T.-F., Dong Y., Ionescu A.M., Rosier R.N., Zuscik M., Schwarz E.M., O’Keefe R.J., Drissi H. (2004). Parathyroid hormone-related peptide (PTHrP) inhibits Runx2 expression through the PKA signaling pathway. Exp. Cell Res..

[27] Vortkamp A., Lee K., Lanske B., Segre G.V., Kronenberg H.M., Tabin C.J. (1996). Regulation of Rate of Cartilage Differentiation by Indian Hedgehog and PTH-Related Protein. Science.

[28] Zelzer E., Glotzer D.J., Hartmann C., Thomas D., Fukai N., Soker S., Olsen B.R. (2001). Tissue specific regulation of VEGF expression during bone development requires Cbfa1/Runx2. Mech. Dev..

[29] Himeno M., Enomoto H., Liu W., Ishizeki K., Nomura S., Kitamura Y., Komori T. (2002). Impaired Vascular Invasion of Cbfa1-Deficient Cartilage Engrafted in the Spleen. J. Bone Miner. Res..

[30] Qin X., Jiang Q., Nagano K., Moriishi T., Miyazaki T., Komori H., Ito K., Von Der Mark K., Sakane C., Kaneko H. (2020). Runx2 is essential for the transdifferentiation of chondrocytes into osteoblasts. PLoS Genet..

[31] Long F., Chung U.-I., Ohba S., McMahon J., Kronenberg H.M., McMahon A.P. (2004). Ihh signaling is directly required for the osteoblast lineage in the endochondral skeleton. Development.

[32] Knauper V., López-Otín C., Smith B., Knight G., Murphy G. (1996). Biochemical Characterization of Human Collagenase-3. J. Biol. Chem..

[33] Sato M., Morii E., Komori T., Kawahata H., Sugimoto M., Terai K., Shimizu H., Yasui T., Ogihara H., Yasui N. (1998). Transcriptional regulation of osteopontin gene in vivo by PEBP2αA/CBFA1 and ETS1 in the skeletal tissues. Oncogene.

[34] Jimenez M.J., Balbin M., Lopez J.M., Alvarez J., Komori T., Lopez-Otin C. (1999). Collagenase 3 is a target of Cbfa1, a transcription factor of the runt gene family involved in bone formation. Mol. Cell. Biol..

[35] Porte D., Tuckermann J., Becker M., Baumann B., Teurich S., Higgins T., Owen M.J., Schorpp-Kistner M., Angel P. (1999). Both AP-1 and Cbfa1-like factors are required for the induction of interstitial collagenase by parathyroid hormone. Oncogene.

[36] Selvamurugan N., Pulumati M.R., Tyson D., Partridge N. (2000). Parathyroid Hormone Regulation of the Rat Collagenase-3 Promoter by Protein Kinase A-dependent Transactivation of Core Binding Factor α1. Biol. Chem..

[37] Hess J., Porte D., Munz C., Angel P. (2001). AP-1 and Cbfa/runt physically interact and regulate parathyroid hormone-dependent MMP13 expression in osteoblasts through a new osteoblast-specific element 2/AP-1 composite element. J. Biol. Chem..

[38] Harada H., Tagashira S., Fujiwara M., Ogawa S., Katsumata T., Yamaguchi A., Komori T., Nakatsuka M. (1999). Cbfa1 Isoforms Exert Functional Differences in Osteoblast Differentiation. J. Biol. Chem..

[39] Yang L., Tsang K.Y., Tang H.C., Chan D., Cheah K.S.E. (2014). Hypertrophic chondrocytes can become osteoblasts and osteocytes in endochondral bone formation. Proc. Natl. Acad. Sci. USA.

[40] Zhou X., Von Der Mark K., Henry S., Norton W., Adams H., De Crombrugghe B. (2014). Chondrocytes Transdifferentiate into Osteoblasts in Endochondral Bone during Development, Postnatal Growth and Fracture Healing in Mice. PLoS Genet..

[41] Park J., Gebhardt M., Golovchenko S., Perez-Branguli F., Hattori T., Hartmann C., Zhou X., Decrombrugghe B., Stock M., Schneider H. (2015). Dual pathways to endochondral osteoblasts: A novel chondrocyte-derived osteoprogenitor cell identified in hypertrophic cartilage. Biol. Open.

[42] Jing Y., Zhou X., Han X., Jing J., Von Der Mark K., Wang J., De Crombrugghe B., Hinton R., Feng J. (2015). Chondrocytes Directly Transform into Bone Cells in Mandibular Condyle Growth. J. Dent. Res..

[43] Mizuhashi K., Ono W., Matsushita Y., Sakagami N., Takahashi A., Saunders T., Nagasawa T., Kronenberg H.M., Ono N. (2018). Resting zone of the growth plate houses a unique class of skeletal stem cells. Nature.

[44] Mizuhashi K., Nagata M., Matsushita Y., Ono W., Ono N. (2019). Growth Plate Borderline Chondrocytes Behave as Transient Mesenchymal Precursor Cells. J. Bone Miner. Res..

[45] Nakashima K., Zhou X., Kunkel G., Zhang Z., Deng J.M., Behringer R.R., de Crombrugghe B. (2002). The Novel Zinc Finger-Containing Transcription Factor Osterix Is Required for Osteoblast Differentiation and Bone Formation. Cell.

[46] Day T.F., Guo X., Garrett-Beal L., Yang Y. (2005). Wnt/β-catenin signaling in mesenchymal progenitors controls osteoblast and chondrocyte differentiation during vertebrate skeletogenesis. Dev. Cell..

[47] Hill T.P., Spater D., Taketo M.M., Birchmeier W., Hartmann C. (2005). Canonical Wnt/β-catenin signaling prevents osteoblasts from differentiating into chondrocytes. Dev. Cell..

[48] Hu H., Hilton M.J., Tu X., Yu K., Ornitz D.M., Long F. (2005). Sequential roles of Hedgehog and Wnt signaling in osteoblast development. Development.

[49] Rodda S.J., McMahon A.P. (2006). Distinct roles for Hedgehog and canonical Wnt signaling in specification, differentiation and maintenance of osteoblast progenitors. Development.

[50] Otto F., Thornell A.P., Crompton T., Denzel A., Gilmour K.C., Rosewell I.R., Stamp G.W., Beddington R.S., Mundlos S., Olsen B.R. (1997). Cbfa1, a Candidate Gene for Cleidocranial Dysplasia Syndrome, Is Essential for Osteoblast Differentiation and Bone Development. Cell.

[51] Komori T., Yagi H., Nomura S., Yamaguchi A., Sasaki K., Deguchi K., Shimizu Y., Bronson R.T., Gao Y.H., Inada M. (1997). Targeted Disruption of Cbfa1 Results in a Complete Lack of Bone Formation owing to Maturational Arrest of Osteoblasts. Cell.

[52] Acampora D., Merlo G.R., Paleari L., Zerega B., Postiglione M.P., Mantero S., Bober E., Barbieri O., Simeone A., Levi G. (1999). Craniofacial, vestibular and bone defects in mice lacking the Distal-less-related gene Dlx5. Development.

[53] Mundlos S., Otto F., Mundlos C., Mulliken J., Aylsworth A., Albright S., Lindhout D., Cole W., Henn W., Knoll J. (1997). Mutations Involving the Transcription Factor CBFA1 Cause Cleidocranial Dysplasia. Cell.

[54] Lee B., Thirunavukkarasu K., Zhou L., Pastore L., Baldini A., Hecht J., Geoffroy V., Ducy P., Karsenty G. (1997). Missense mutations abolishing DNA binding of the osteoblast-specific transcription factor OSF2/CBFA1 in cleidocranial dysplasia. Nat. Genet..

[55] Qin X., Jiang Q., Miyazaki T., Komori T. (2018). Runx2 regulates cranial suture closure by inducing hedgehog, Fgf, Wnt and Pthlh signaling pathway gene expressions in suture mesenchymal cells. Hum. Mol. Genet..

[56] Xiao G., Jiang D., Gopalakrishnan R., Franceschi R.T. (2002). Fibroblast Growth Factor 2 Induction of the Osteocalcin Gene Requires MAPK Activity and Phosphorylation of the Osteoblast Transcription Factor, Cbfa1/Runx2. J. Biol. Chem..

[57] Park O.-J., Kim H.-J., Woo K.M., Baek J.-H., Ryoo H.-M. (2010). FGF2-activated ERK Mitogen-activated Protein Kinase Enhances Runx2 Acetylation and Stabilization. J. Biol. Chem..

[58] Gaur T., Lengner C., Hovhannisyan H., Bhat R.A., Bodine P.V.N., Komm B.S., Javed A., van Wijnen A.J., Stein J.L., Stein G.S. (2005). Canonical WNT Signaling Promotes Osteogenesis by Directly Stimulating Runx2 Gene Expression. J. Biol. Chem..

[59] Kawane T., Komori H., Liu W., Moriishi T., Miyazaki T., Mori M., Matsuo Y., Takada Y., Izumi S., Jiang Q. (2014). Dlx5 and Mef2 Regulate a Novel Runx2 Enhancer for Osteoblast-Specific Expression. J. Bone Miner. Res..

[60] Lee M.-H., Kim Y.-J., Yoon W.-J., Kim J.-I., Kim B.-G., Hwang Y.-S., Wozney J.M., Chi X.-Z., Bae S.-C., Choi K.-Y. (2005). Dlx5 Specifically Regulates Runx2 Type II Expression by Binding to Homeodomain-response Elements in the Runx2 Distal Promoter. J. Biol. Chem..

[61] Krishnan V., Moore T.L., Ma Y.L., Helvering L.M., Frolik C.A., Valasek K.M., Ducy P., Geiser A.G. (2003). Parathyroid Hormone Bone Anabolic Action Requires Cbfa1/Runx2-Dependent Signaling. Mol. Endocrinol..

[62] Yoshida C.A., Komori H., Maruyama Z., Miyazaki T., Kawasaki K., Furuichi T., Fukuyama R., Mori M., Yamana K., Nakamura K. (2012). SP7 Inhibits Osteoblast Differentiation at a Late Stage in Mice. PLoS ONE.

[63] Komori T. (2020). Functions of Osteocalcin in Bone, Pancreas, Testis, and Muscle. Int. J. Mol. Sci..

[64] Pratap J., Galindo M., Zaidi K., Vradii D., Bhat B.M., A Robinson J., Choi J.-Y., Komori T., Stein J.L., Lian J.B. (2003). Cell growth regulatory role of Runx2 during proliferative expansion of preosteoblasts. Cancer Res..

[65] Kawane T., Qin X., Jiang Q., Miyazaki T., Komori H., Yoshida C.A., Matsuura-Kawata V.K.D.S., Sakane C., Matsuo Y., Nagai K. (2018). Runx2 is required for the proliferation of osteoblast progenitors and induces proliferation by regulating Fgfr2 and Fgfr3. Sci. Rep..

[66] Komori T. (2010). Regulation of bone development and extracellular matrix protein genes by RUNX2. Cell Tissue Res..

[67] Adhami M.D., Rashid H., Chen H., Javed A. (2014). Runx2 activity in committed osteoblasts is not essential for embryonic skeletogenesis. Connect. Tissue Res..

[68] Qin X., Jiang Q., Komori H., Sakane C., Fukuyama R., Matsuo Y., Ito K., Miyazaki T., Komori T. (2021). Runt-related transcription factor-2 (Runx2) is required for bone matrix protein gene expression in committed osteoblasts in mice. J. Bone Miner. Res..

[69] Mak K.K., Bi Y., Wan C., Chuang P.-T., Clemens T., Young M., Yang Y. (2008). Hedgehog Signaling in Mature Osteoblasts Regulates Bone Formation and Resorption by Controlling PTHrP and RANKL Expression. Dev. Cell.

[70] Riddle R.C., Diegel C.R., Leslie J.M., Van Koevering K.K., Faugere M.-C., Clemens T.L., Williams B.O. (2013). Lrp5 and Lrp6 Exert Overlapping Functions in Osteoblasts during Postnatal Bone Acquisition. PLoS ONE.

[71] Baek W.-Y., Lee M.-A., Jung J.W., Kim S.-Y., Akiyama H., De Crombrugghe B., Kim J.-E. (2009). Positive regulation of adult bone formation by osteoblast-specific transcription factor osterix. J. Bone Miner. Res..

[72] Baek W.-Y., de Crombrugghe B., Kim J.-E. (2010). Postnatally induced inactivation of Osterix in osteoblasts results in the reduction of bone formation and maintenance. Bone.

[73] Enomoto H., Shiojiri S., Hoshi K., Furuichi T., Fukuyama R., Yoshida C.A., Kanatani N., Nakamura R., Mizuno A., Zanma A. (2003). Induction of osteoclast differentiation by Runx2 through receptor activator of nuclear factor-kappa B ligand (RANKL) and osteoprotegerin regulation and partial rescue of osteoclastogenesis in Runx2^−/−^ mice by RANKL transgene. J. Biol. Chem..

[74] Moriishi T., Ozasa R., Ishimoto T., Nakano T., Hasegawa T., Miyazaki T., Liu W., Fukuyama R., Wang Y., Komori H. (2020). Osteocalcin is necessary for the alignment of apatite crystallites, but not glucose metabolism, testosterone synthesis, or muscle mass. PLoS Genet..

[75] Diegel C.R., Hann S., Ayturk U.M., Hu J.C.W., Lim K.-E., Droscha C.J., Madaj Z.B., Foxa G.E., Izaguirre I., Core V.V.A.T. (2020). An osteocalcin-deficient mouse strain without endocrine abnormalities. PLoS Genet..

[76] Catheline S.E., Hoak D., Chang M., Ketz J.P., Hilton M.J., Zuscik M.J., Jonason J.H. (2019). Chondrocyte-Specific RUNX2 Overexpression Accelerates Post-traumatic Osteoarthritis Progression in Adult Mice. J. Bone Miner. Res..

[77] Kamekura S., Kawasaki Y., Hoshi K., Shimoaka T., Chikuda H., Maruyama Z., Komori T., Sato S., Takeda S., Karsenty G. (2006). Contribution of runt-related transcription factor 2 to the pathogenesis of osteoarthritis in mice after induction of knee joint instability. Arthritis Care Res..

[78] Hirata M., Kugimiya F., Fukai A., Saito T., Yano F., Ikeda T., Mabuchi A., Sapkota B.R., Akune T., Nishida N. (2012). C/EBPβ and RUNX2 cooperate to degrade cartilage with MMP-13 as the target and HIF-2α as the inducer in chondrocytes. Hum. Mol. Genet..

[79] Liao L., Zhang S., Gu J., Takarada T., Yoneda Y., Huang J., Zhao L., Oh C.-D., Li J., Wang B. (2017). Deletion of Runx2 in Articular Chondrocytes Decelerates the Progression of DMM-Induced Osteoarthritis in Adult mice. Sci. Rep..

